# Comparison of the recommendations of the AAPM TG‐51 and TG‐51 addendum reference dosimetry protocols

**DOI:** 10.1002/acm2.12110

**Published:** 2017-06-02

**Authors:** Travis J. McCaw, Min‐Sig Hwang, Si Young Jang, M. Saiful Huq

**Affiliations:** ^1^ Department of Human Oncology University of Wisconsin Madison WI USA; ^2^ Department of Radiation Oncology University of Pittsburgh Cancer Institute and UPMC CancerCenter Pittsburgh PA USA

**Keywords:** photon beam reference dosimetry, TG‐51 addendum

## Abstract

This work quantified differences between recommendations of the TG‐51 and TG‐51 addendum reference dosimetry protocols. Reference dosimetry was performed for flattened photon beams with nominal energies of 6, 10, 15, and 23 MV, as well as flattening‐filter free (FFF) beam energies of 6 and 10 MV, following the recommendations of both the TG‐51 and TG‐51 addendum protocols using both a Farmer^®^ ionization chamber and a scanning ionization chamber with calibration coefficients traceable to absorbed dose‐to‐water (*D*_w_) standards. Differences in *D*_w_ determined by the two protocols were 0.1%–0.3% for beam energies with a flattening filter, and up to 0.2% and 0.8% for FFF beams measured with the scanning and Farmer^®^ ionization chambers, respectively, due to *k*_Q_ determination, volume‐averaging correction, and collimator jaw setting. Combined uncertainty was between 0.91% and 1.2% (*k *= 1), varying by protocol and detector.

**Abbreviation**
*D*_w_: absorbed dose‐to‐water
FFF: flattening‐filter free
*P*_leak_: leakage ionization correction
*P*_rp_: volume‐averaging correction
TG‐51: Task Group 51 report
TG‐51 addendum: addendum to Task Group 51 report

## 1 INTRODUCTION

In 1999 the AAPM Task Group 51 report (TG‐51) was published, establishing an *D*_w_‐based protocol for clinical reference dosimetry of photon and electron beams in place of the previously recommended air kerma‐based protocol.^1^ According to IROC Houston, 99.8% of North American institutions with compliant machines have implemented TG‐51 for clinical reference dosimetry.^2^ To address advances since the publication of TG‐51, the AAPM published an addendum to this report (TG‐51 addendum) in 2014.^3^ TG‐51 addendum updates *k*_Q_ values using Monte Carlo simulations, revises recommendations regarding the use of a lead foil for the determination of *k*_Q_ values to address FFF beams and reduce the potential for error, introduces correction factors to account for averaging of the detector signal over the sensitive volume of the ionization chamber (*P*_rp_) and contributions from leakage current (*P*_leak_), and recommends best practices for protocol implementation to minimize uncertainty. Papageorgiou et al.^4^ found that the updates to the *k*_Q_ values had an insignificant impact on accelerator output, but did not address the remaining updates in TG‐51 addendum. In the current work, accelerator output determined following TG‐51 and TG‐51 addendum using both a 0.6 cm^3^ PTW N30013 Farmer^®^ ionization chamber (Freiburg, Germany) and a 0.053 cm^3^ Exradin A1SL scanning ionization chamber (Standard Imaging, Inc., Middleton, WI, USA) was compared.

## 2 METHODS

Reference dosimetry for photon beams with nominal energies of 6 MV (6X, 6X‐SRS, 6XFFF), 10 MV (10X, 10XFFF), 15 MV (15X), and 23 MV (23X) was performed following the recommendations of TG‐51 and TG‐51 addendum. The 6XFFF and 10XFFF energies do not have flattening filters, and the 6X‐SRS energy has a thin filter capable of flattening fields up to 15 × 15 cm^2^. Measurements of the 6X‐SRS and 23X energies were completed on a Trilogy^®^ linear accelerator (Varian Medical Systems, Inc., Palo Alto, CA, USA), while measurements of the remaining energies were completed on a Varian TrueBeam™ STx linear accelerator. A 0.6 cm^3^ PTW N30013 Farmer^®^ ionization chamber and a 0.053 cm^3^ Exradin A1SL scanning ionization chamber, both with ADCL *N*_D,w_ calibration coefficients, were used with an ADCL‐calibrated PTW UNIDOS electrometer for measurements in a Blue Phantom 3D scanning water tank (IBA Dosimetry, Schwarzenbruck, Germany). PDD, inline, and crossline profiles were measured with a 0.13 cm^3^ IBA CC13 scanning ionization chamber.

Prior to measurements, ionization chambers were allowed to thermally equilibrate with the water for 30 min. A bias voltage of −300 V was applied to the ionization chamber, and a dose of 10 Gy was delivered to the ionization chamber to stabilize the measured ionization current. The leakage current was then nullified. Following each change in bias voltage, a dose of 10 Gy was delivered to the ionization chamber to stabilize the measured ionization current. Reference dose measurements were completed at a SAD of 100 cm using a vertical beam orientation. Five consecutive measurements of 100 MU were averaged for each measurement of raw ionization. The two‐voltage technique was used to determine the recombination correction with bias voltages of −300 V and −100 V. As discussed in Section 3, the validity of the two‐voltage technique was verified with measurements of Jaffé plots.

In this work the TG‐51 procedure used a lead foil for *%dd*(10)_x_ measurements for energies greater than 10 MV (i.e., 15 MV and 23 MV), determined *k*_Q_ from *%dd*(10)_x_ using data for the PTW N30001 and Exradin A1 ionization chambers from table 1 of Ref. 1, and set the collimator jaws to a 10 × 10 cm^2^ field according to the digital readout. Conversely, the TG‐51 addendum procedure in this work used a lead foil for *%dd*(10)_x_ measurements for the FFF beams, used the interim measure (i.e., Eq. 15 of Ref. 1) to calculate *%dd*(10)_x_ for energies greater than 10 MV, determined *k*_Q_ from *%dd*(10)_x_ using Table 1 of Ref. 3, set the collimator jaws to define a 10 × 10 cm^2^ radiation field, and applied *P*_leak_ and *P*_rp_ corrections to the raw ionization reading. The fluence incident on the ionization chamber, and therefore the total ionization produced within the chamber, is dependent on the size of the radiation field. Consequently, deviation from the specified 10 × 10 cm^2^ field size introduces measurement uncertainty, which TG‐51 addendum emphasizes must be considered. The collimator jaw settings for a 10 × 10 cm^2^ radiation field in the TG‐51 addendum procedure were determined by measurement of the full width at half maximum of inline and crossline profile scans with the CC13 ionization chamber. Between each adjustment of the collimator position, the jaws were first programmed to a 15 × 15 cm^2^ field to prevent hysteresis and minimize mechanical slop. *P*_leak_ was determined using a one‐minute integration of ionization current in the absence of a radiation field with a bias voltage of −300 V applied to the ionization chamber. *P*_rp_ was determined as the product of *k*_vol,in_ and *k*_vol,cr_:

(1) $$k_{\mathrm{vol},\mathrm{in}}=\frac{\int \;\;\;\int_{A}^{}w\left(0,y\right)dy}{\int \;\;\;\int_{A}^{}w\left(0,y\right)OAR\left(0,y\right)dy},k_{\mathrm{vol},\mathrm{cr}}=\frac{\int \;\;\;\int_{A}^{}w\left(x,0\right)dx}{\int \;\;\;\int_{A}^{}w\left(x,0\right)OAR\left(x,0\right)dx}$$

where *OAR*(*x*,*y*) is the inline (*y*)/crossline (*x*) profile normalized to the central axis, and

(2) $$w\left(x,y\right)=\sqrt{R^{2}-x^{2}}$$

where *R* is the radius of the collecting volume.

**Table 1:** PTW N30013 Farmer^®^ ionization chamber results

Energy	6X	6X‐SRS	10X	15X	23X	6XFFF	10XFFF
${\left(D_{w}/\mathrm{MU}\right)}_{\mathrm{TG}51}^{\mathrm{addendum}}$	1.001	0.999	1.002	0.999	1.001	1.005	1.008
${\left(k_{Q}\right)}_{\mathrm{TG}51}^{\mathrm{addendum}}$	1.000	1.001	1.001	1.002	1.002	0.999	1.000
*P*_rp_	1.000	0.998	1.001	0.996	0.999	1.004	1.006
${\left(M_{\mathrm{raw}}\right)}_{\mathrm{TG}51}^{\mathrm{addendum}}$	1.001	1.000	1.001	1.001	1.000	1.002	1.002
Digital readout for 10 × 10 cm^2^ radiation field	9.9 × 10.2	9.9 × 10.0	10.0 × 10.2	9.9 × 10.2	9.9 × 10.0	10.0 × 10.3	10.1 × 10.4

## 3 RESULTS AND DISCUSSION

Comparisons of reference dosimetry following the recommendations of TG‐51 and TG‐51 addendum using the PTW N30013 and Exradin A1SL ionization chambers are shown in Tables 1 and 2, respectively. The parameters not shown in these tables, namely *P*_ion_, *P*_pol_, *P*_elec_, and *P*_TP_, are identical between the two protocols. For the Exradin A1SL ionization chamber, differences in *D*_w_/MU between the two protocols ranged between 0.1% and 0.3%. For the PTW N30013 ionization chamber, differences in *D*_w_/MU ranged between 0.1% and 0.2% for beam energies with a flattening filter, and 0.5% and 0.8% for FFF beam energies.

**Table 2:** Exradin A1SL scanning ionization chamber results

Energy	6X	6X‐SRS	10X	15X	23X	6XFFF	10XFFF
${\left(D_{w}/\mathrm{MU}\right)}_{\mathrm{TG}51}^{\mathrm{addendum}}$	0.999	0.997	0.998	0.998	0.999	1.001	0.998
${\left(k_{Q}\right)}_{\mathrm{TG}51}^{\mathrm{addendum}}$	0.998	0.998	0.996	0.998	0.999	0.998	0.996
*P*_rp_	1.000	1.000	1.000	0.999	1.000	1.001	1.000
${\left(M_{\mathrm{raw}}\right)}_{\mathrm{TG}51}^{\mathrm{addendum}}$	1.002	0.999	1.001	1.001	1.001	1.002	1.003

Differences in the output determined using the two protocols were due to *k*_Q_ determination, use of the *P*_rp_ correction, and collimator jaw setting. The ratios of *k*_Q_ determined using the two protocols for the PTW N30013 and Exradin A1SL ionization chambers ranged from 0.999–1.002 and 0.996–0.999, respectively. TG‐51 addendum states that differences of up to 0.5% were found between the new and original *k*_Q_ values.^3^ Furthermore, the ionization chambers used in this work were not included in TG‐51, which can result in a *k*_Q_ assignment uncertainty of up to 0.5%.^3^ Therefore, the differences in *k*_Q_ between the two protocols observed in this work are within expected variations for ionization chambers that were not listed in TG‐51. For the smaller‐volume Exradin A1SL chamber, *P*_rp_ did not exceed 0.1%, while *P*_rp_ values of up to 0.4% and 0.6% were measured for beam energies with and without a flattening filter, respectively, for the PTW N30013 chamber. For comparison, Kry et al.^5^ determined that a volume‐averaging correction of 0.2% was necessary for measurements of 6XFFF and 10XFFF beam energies from a TrueBeam™ accelerator using a Farmer‐type chamber. Whereas *P*_rp_ was determined by averaging over inline and crossline dose profile scans in the current work, Kry et al.^5^ used a 1D average over an inline dose profile measured using film. The difference between a 1D and 2D average to determine *P*_rp_ is minimal; in the current work, the largest correction from averaging over the crossline profile was less than 0.05%. The digital readout for a 10 × 10 cm^2^ radiation field is shown in Table 1 for each beam energy. Measured ionization differed up to 0.2% and 0.3% between 10 × 10 cm^2^ fields defined according to the digital readout (i.e., TG‐51 procedure) or dose profile scans (i.e., TG‐51 addendum procedure) for the PTW N30013 and Exradin A1SL chambers, respectively.

Differences in *D*_w_/MU measured with the two ionization chambers following a given reference dosimetry protocol are shown in Table 3. Differences of up to 0.80% between the PTW N30013 and Exradin A1SL ionization chambers were observed for TG‐51, while the largest difference for TG‐51 addendum was 0.40%. As discussed below, these differences are all within the estimated measurement uncertainty (*k* = 1).

**Table 3:** Percentage difference in *D*_w_/MU measured with each ionization chamber

Energy	6X	6X‐SRS	10X	15X	23X	6XFFF	10XFFF
TG‐51	0.50	0.03	0.47	0.11	0.13	0.57	0.80
TG‐51 addendum	0.40	0.11	0.22	0.06	0.19	0.38	0.28

Uncertainty estimates for both reference dosimetry protocols performed with each ionization chamber are shown in Table 4 as percent standard uncertainties. The combined uncertainty estimate assumes that all uncertainties are uncorrelated. The SSD for both protocols was measured using the front pointer with an estimated uncertainty of 0.5 mm. The methodology from AAPM Task Group 106 was used to define the water surface with an estimated uncertainty of 0.5 mm.^6^ Based on the constancy of repeated profile measurements, an uncertainty of 0.5 mm in the field size setting was assumed for TG‐51 addendum, while an uncertainty of 4 mm was assumed for TG‐51 based on established tolerances for jaw positioning.^7^ The uncertainties in Table 4 are based on measured differences in ionization due to the estimated jaw positioning uncertainty. Uncertainty in the temperature‐pressure correction was determined based on observed variations in the temperature and pressure of 0.5°C and 0.33 kPa, respectively, over the course of measurements. No consideration was made for humidity, so the uncertainty from Table 4 was assumed based on a humidity range of 20%–80%. The uncertainty in charge measurement is based on maximum differences between repeated ionization measurements with an applied bias of −300 V, with a change in bias voltage between measurements. Monthly intercomparisons between the reference ionization chambers and a third reference‐class ionization chamber indicated stability of the detectors within ±0.1%. A dose of 10 Gy was delivered to each ionization chamber after changes in applied bias, limiting the short‐term drift in ionization chamber response to less than 0.1%.^8^ Following nullification of the leakage current, the measured leakage over a one‐minute integration in the absence of radiation was 0 fC; therefore, no correction was applied for leakage, and an uncertainty of 0.1% was assumed. Measurements of Jaffé plots following the methodology of McEwen^8^ verified the accuracy of the two‐voltage technique for the determination of *P*_ion_ within 0.1%. The uncertainty in the polarity correction was determined from the uncertainty in charge measurement. The uncertainty in the linac stability was taken as 0.05%.^3^ Based on the repeatability of profiles measured with different detectors, the uncertainty in *P*_rp_ was estimated as 0.1% for TG‐51 addendum. For TG‐51, which does not recommend the use of a volume‐averaging correction, the uncertainty estimates are based on the maximum *P*_rp_ values determined with TG‐51 addendum. The uncertainty in the *k*_Q_ factor was taken as 0.4% and 0.5% for TG‐51 addendum and TG‐51,^3^ respectively. The ionization chambers used in this work are not listed in TG‐51, so an uncertainty of 0.5% in the assignment of *k*_Q_ was estimated based on the use of *k*_Q_ values for chambers with similar construction.^1^ The use of the interim method for *k*_Q_ determination of energies greater than 10 MV in the TG‐51 addendum procedure contributes a *k*_Q_ assignment uncertainty of 0.2%.^3^ As shown in Table 4, the combined uncertainty (*k* = 1) is 0.2% to 0.3% lower when following TG‐51 addendum, due primarily to improved accuracy in *k*_Q_ assignment, use of a volume‐averaging correction, and verification of the field‐size setting.

**Table 4:** Uncertainty budget with all values given as percent standard uncertainties

Parameter	TG‐51		TG‐51 addendum	
	N30013	A1SL	N30013	A1SL
SSD setting	0.10	0.10	0.10	0.10
Depth setting	0.25	0.25	0.25	0.25
Field‐size setting	0.20	0.20	0.05	0.05
Charge measurement	0.10	0.30	0.10	0.30
*P*_TP_ correction	0.18	0.18	0.18	0.18
Humidity	0.15	0.15	0.15	0.15
^60^Co *N*_D,w_	0.65	0.65	0.65	0.65
*k*_Q_ factor	0.50	0.50	0.40	0.40
Assignment of *k*_Q_ factor	0.50	0.50	0.20	0.20
Stability of reference chamber	0.10	0.10	0.10	0.10
*P*_pol_	0.10	0.30	0.10	0.30
*P*_ion_	0.10	0.10	0.10	0.10
Pre‐irradiation history	0.10	0.10	0.10	0.10
Leakage current	0.10	0.10	0.10	0.10
Linac stability	0.05	0.05	0.05	0.05
*P*_elec_	0.10	0.10	0.10	0.10
*P*_rp_	0.60	0.10	0.10	0.10
Combined (*k* = 1)	1.2	1.2	0.91	0.99

Procedurally, the greatest difference between the two protocols is the measurement of inline and crossline dose profiles to correct for dose averaging over the volume of the ionization chamber, which may require a different water phantom than is normally used. Additionally, if profile scanning is used to measure the inline and crossline dose profiles, then a scanning ionization chamber should be used. To avoid the need to setup multiple detectors, adopters of TG‐51 addendum may prefer to use a reference‐class scanning ionization chamber, such as the Exradin A1SL chamber employed in this work, to perform reference dosimetry. However, as stated in TG‐51 addendum, only reference‐class ionization chambers should be used for reference dosimetry. Apart from the measurement of inline and crossline dose profiles, the implementation of TG‐51 addendum requires only the use of different reference data (i.e., *k*_Q_ determination) and adjustments to existing procedures (i.e., use of a lead foil). For institutions that employ in‐house spreadsheets for the calculation of *D*_w_/MU from the data collected during reference dosimetry, the necessary changes to these spreadsheets for the implementation of TG‐51 addendum should be carefully verified. During the initial implementation of TG‐51 addendum, reference dosimetry should be completed following both protocols to quantify the impact the change in reference dosimetry protocol will have on dosimetric output. Although the expected change in output is less than 1% as shown in this work, physicians should be notified of the change since the dose delivered to patients will be impacted.

## 4 CONCLUSIONS

In this work differences between the TG‐51 and TG‐51 addendum reference dosimetry protocols were quantified. Differences of up to 0.8% in the *D*_w_/MU were measured between the protocols, with a measurement uncertainty of 0.91%–1.2% (*k *= 1). For reference dosimetry of FFF beam energies with a 0.6 cc Farmer‐type ionization chamber, volume‐averaging corrections of up to 0.6% were measured. Failure to verify the dimensions of the radiation field resulted in measurement differences of up to 0.3%.

## CONFLICT OF INTEREST

The authors declare no conflict of interest.
